# Vault RNAs: hidden gems in RNA and protein regulation

**DOI:** 10.1007/s00018-020-03675-9

**Published:** 2020-10-15

**Authors:** Jens Claus Hahne, Andrea Lampis, Nicola Valeri

**Affiliations:** 1 Division of Molecular Pathology, The Institute of Cancer Research, London, UK; 2 Centre for Evolution and Cancer, The Institute of Cancer Research, London, UK; 3 Department of Medicine, The Royal Marsden NHS Foundation Trust, London, UK

**Keywords:** Vault RNA, Non-coding RNAs, Vault ribonucleoprotein complex

## Abstract

Non-coding RNAs are important regulators of differentiation during embryogenesis as well as key players in the fine-tuning of transcription and furthermore, they control the post-transcriptional regulation of mRNAs under physiological conditions. Deregulated expression of non-coding RNAs is often identified as one major contribution in a number of pathological conditions. Non-coding RNAs are a heterogenous group of RNAs and they represent the majority of nuclear transcripts in eukaryotes. An evolutionary highly conserved sub-group of non-coding RNAs is represented by vault RNAs, named since firstly discovered as component of the largest known ribonucleoprotein complexes called “vault”. Although they have been initially described 30 years ago, vault RNAs are largely unknown and their molecular role is still under investigation. In this review we will summarize the known functions of vault RNAs and their involvement in cellular mechanisms.

## Introduction

Non-coding RNAs represent the vast majority of transcriptional product of the human genome [1, 2]. The family of non-coding RNAs is composed of 19 different classes; among them transfer RNAs (tRNAs), tRNA-derived RNA fragments (tRFs), ribosomal RNAs (rRNAs), small nucleolar RNAs (snoRNAs), endogenous small interfering RNAs (endo-siRNAs), sno-derived RNAs (sdRNAs), transcription initiation RNAs (tiRNAs), miRNA-offset-RNAs (moRNAs), circular RNAs (circRNAs), vault RNAs, microRNAs (miRNAs), small interfering RNAs (siRNAs), small nuclear RNAs (snRNAs), extracellular RNAs (exRNAs), piwi-interacting RNAs (piRNAs), small Cajal body RNAs (scaRNAs), transcribed-ultraconserved regions (t-UCRs), long intergenic non-coding RNAs (lincRNAs), and long non-coding RNAs (lncRNAs) [3–25]. The role and function of tRNAs, rRNAs, microRNAs and lncRNAs, in particular, have been well examined both under physiological and pathological conditions [26]. In general, non-coding RNAs control all levels of genes’ regulation in eukaryotes, including the control of chromosome dynamics, splicing, RNA editing, translational inhibition and mRNA degradation [26]. Even transcription itself may be regulated by non-coding RNAs as outlined in several reports [27–29]. This is achieved on one hand, by control of chromosome dynamics and modifications and on the other hand, by regulation of RNA polymerase II activity. Therefore, non-coding RNAs are involved in regulation of accessibility of DNA sequences for the transcription machinery, as well as in modulation of the transcription rate of RNA polymerase II [30–34]. Furthermore, splicing of pre-mRNA transcripts, post-transcriptional regulation of expression rate as well as translation of mRNAs in cytoplasm and regulation of mRNA half-life are under control of non-coding RNAs [26, 27, 35]. In addition, some non-coding RNAs are known to be involved in intercellular communication and cell regulation [36, 37]; whereas, others are part of the antiviral defence by stimulating immune response and activating RNA interference pathway [38, 39].

In contrast, the molecular functions of vault RNAs are still not completely clear even after more than 30 years since their discovery [40, 41]. With a length between 88 and 140 nucleotides vault RNAs are longer than miRNAs, but they are still included as members of the short non-coding RNA group [41, 42].

In humans, four vault RNAs are encoded on chromosome 5q31 in two different loci. The VTRNA-1 locus (located between zinc-finger matrin-type 2 gene and proto-cadherin cluster) contains the genetic information for three vault RNAs (vault RNA1-1, vault RNA1-2 and vault RNA1-3) and VTRNA-2 locus (located between the genes coding for transforming growth factor beta 1 and SMAD family member 5) codes for vault RNA2-1 also known as pre-miR-886 [43–45]. All vault RNA genes are under control of a polymerase III type 2 promoter and they contain a box A and box B motif normally found in tRNA genes [41, 42]. Nevertheless, the promoters of the two vault RNA loci are not identical; therefore, expression patterns of the vault RNA genes are different [42]. Furthermore, epigenetic modifications such as promoter methylation are important regulators for vault RNAs expression especially for the VTRNA-2 gene [46, 47]. The distant regulatory elements of the VTRNA-1 promoter are characterized by differential CpG accessibility and this might be a hint for a cell-type-specific expression of the three vault RNAs under control of this promoter [48]. The internal promoter sequences box A and box B present in VTRNA-1 and VTRNA-2 enable binding of transcription factors TFIIIC and TFIIIB which facilitate polymerase III binding to the transcription starting site [49]. Vault RNAs transcription is also under control of cAMP response (CRE)- and tetradecanoyl-phorbol acetate response (TRE)-like elements [41, 42]. These elements represent binding sites for the transcription factors CREB and AP-1, respectively, which adapt key cellular processes such as differentiation, proliferation and survival to nutrient, growth factor and stress signaling [50, 51]. This could explain the observed differential vault RNA transcription rate upon viral infection, starvation and cancer [45, 52–54]. Furthermore, the short half-life time observed of around 1 h makes vault RNAs suitable as signaling molecules that quickly respond to stimuli [55, 56].

**Fig. 1:**
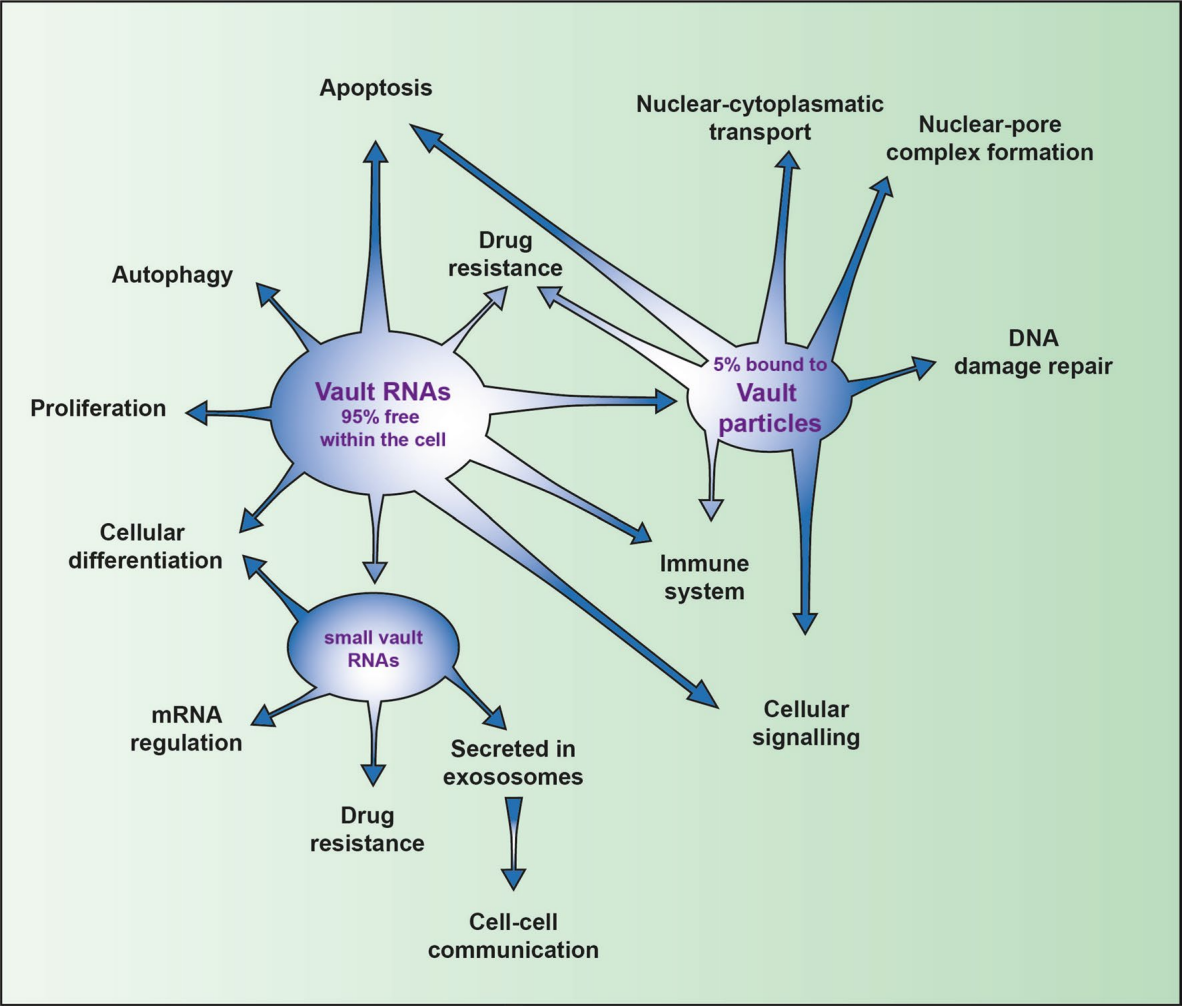
Vault RNAs are involved in different cellular processes and interactions. The vast majority (95%) of vault RNAs are not associated with the vault ribonucleoprotein complex; they are involved in regulation of important cellular pathways like cell death (intrinsic and extrinsic apoptosis; autophagy), proliferation, cellular differentiation, mRNA regulation and cell–cell communication. Besides this, vault RNAs influence the immune reaction and especially in cancer, they have an important role in rendering cells resistance to drug treatment

Vault RNAs were first identified as a component of vault particles [40] but most of the vault RNAs (around 95%) are not associated with these particles and therefore, vault RNAs are most probably also involved in other cellular processes and interactions [57, 58] (Fig. 1).

## Vault particles

Vault particles are the largest known ribonucleoprotein complexes in eukaryotes [59, 60]. Structure and protein composition of vaults are highly conserved and present in high number (10,000–100,000/cell) in different species [43, 61–64]. Therefore, it is very likely that vault particles might play fundamental roles in eukaryotic cells [42]. Some reports connect the vault particles complex to a plethora of mechanisms that include: nuclear-pore complex formation, nuclear–cytoplasmic transport, cellular signaling, innate immune response, apoptosis resistance, DNA damage repair and development of multidrug resistance in cancer cells [43, 65–72] (Fig. 1).

The main component of vault particles is the major vault protein that is sufficient itself for the structural conformation characteristic of the vault particles [73–77]. The major vault protein has no homology to any other protein known, but it is highly conserved among different species (around 90% identity between mammalians and around 60% with lower organisms) [75, 78]. Interestingly, the major vault protein contains two Ca^2+^ binding sites at the N-terminal end which are necessary for correct folding and particle assembly but also to interact with other proteins like PTEN, thus connecting the vault particles to cellular signaling pathways [79]. Beside the major vault protein, two other proteins are present in the vaults particles; the poly-(adenosine-diphosphate ribose) polymerase—a member of the PARP family—and the telomerase-associated protein 1 (TEP1) [80–82]. In these vault particles, the vault RNAs are associated with the caps [43, 57, 75, 83]. The vast majority (around 90%) of vault particles in unstressed cells are located in the cytoplasm but vault particles are also found to be associated with the nuclear membrane. The distribution of vault particles varies in response to external stimuli and rapidly react towards extracellular changes with translocation to different subcellular compartments. Furthermore, under pathological conditions like cancer, a higher amount of vault particles are associated with the nuclear membrane and up to 5% of them are found within the nucleus [68, 84–88]. Based on this observation and the barrel-like structure of the vault particles, the hypothesis exist that vault particles have an important role in mediating shuttle processes between cytoplasm and nucleus, including nuclear import of tumor-suppressors like PTEN, nuclear hormone receptors as well as drug export. It is speculated that some of the cargos transported in vault particles are bound to the vault RNAs present in these complexes [66, 67, 89–91]. But up to now, the role of vault particles as transporter is still under discussion and further investigation is urgently needed because most of these studies used either immunoprecipitation of signaling complexes or yeast two hybrid systems, and it cannot be excluded that the found interaction with vault particles and vault RNAs occurred accidentally and is without any biological sense. A verification of vault particles as transporter in humans under physiological and/or pathological conditions (e.g., tumor) is still missing.

## Studies on vault particles using knock-out mice

The role of major vault protein and vault particles has been addressed in relevant mice knock-out models [92–94]. In TEP1 knock-out mice vault particles were still present, but inside these vault particles, no vault RNAs have been found. Therefore, it was concluded that TEP1 is absolutely required for a stable association of vault RNAs with the vault complex [93]. TEP1 knock-out mice as well as major vault protein knock-out mice are viable, healthy and display no obvious abnormalities [93, 94]. The major vault protein knock-out mice express no vaults particles as expected and have been used in different studies to examine the role of vault particles [78, 84, 94, 95]. Surprisingly, embryonic stem cells and bone marrow cells derived from major vault protein knock-out mice showed no change in sensitivity to drugs when compared to wild-type mice cells. In addition, the activities of the multidrug resistance-related transporters P-glycoprotein, multidrug resistance-associated protein and breast cancer resistance protein were not altered in vault-deficient cells ruling out the possibility that these proteins compensate for the loss of vaults. Also, the response towards doxorubicin treatment was the same in major vault protein knock-out and wild-type mice in in vivo experiments [94]. These observations lead to the conclusion that at least in mice, vaults are not directly involved in drug resistance [78, 84]. In another study, the major vault protein knock-out mice were used to address the role of vaults in regard to dendritic cells. Development and function of dendritic cells, derived from mononuclear bone marrow cells, appeared normal in knock-out mice. In-vivo immunization assays showed that neither T-cell-mediated immune response nor T-cell-dependent humoral response were affected by major vault protein knock-out, indicating intact antigen-presenting and migration capacities of dendritic cells. Obviously, in mice vault particles are not required for primary dendritic cell functions [95]. This observation is in contrast with findings in humans where major vault protein and vault particles are up-regulated during the development of human dendritic cells. Moreover, major vault protein-specific antibodies, presumably interfering with the function of major vault protein or vaults, resulted in reduced expression levels of dendritic cell markers, co-stimulatory molecules and decreased capacity to induce T-cell proliferative and interferon-ɣ-releasing responses [96]. Recently, the major vault protein was identified as a suppressor for NF-κB signaling in macrophages [97]. Global as well as myeloid-specific major vault protein gene knock-out intensified high-fat diet-induced obesity, insulin resistance, hepatic steatosis and atherosclerosis in mice via NF-κB signaling pathway. Furthermore, increased macrophage infiltration and inflammatory responses in the microenvironments have been observed [97]. Another study used peripheral blood mononuclear cells (PBMCs) from major vault protein knock-out mice and evaluated an essential role of major vault protein for the induction of early antiviral cytokines (like IL-6 and IL-8) in the context of double-stranded RNA- or virus-induced pro-inflammatory response [98]. In the following sections, we will focus on the role of vault particles and vault RNAs in humans.

## Vault RNAs, vault particles and drug resistance

One of the roles of the vault particles is the contribution to mediate drug resistance mechanisms by transporting the drugs from their intracellular targets to the extracellular compartment and also in drug sequestration [78]. In an elegant experiment, expression of major vault proteins was prevented by a siRNA approach in human bladder cancer cells under doxycycline treatment. This resulted in inhibition of cytosolic doxorubicin sequestration in perinuclear lysosomes and enhanced accumulation of the drug in the nucleus as well as increased cytotoxicity [99]. Based on the fact that nuclear PTEN is involved in the maintenance of chromosomal stability [100], its nuclear transportation by vaults particles could also play a role in drug resistance mechanisms by counteracting drug-induced DNA damage [101].

In most cell lines, vault RNA1-1 has the highest expression level of all vault RNA transcripts [102]. In multidrug-resistant cells, the level of vault RNA1-1 is not altered but expression rate of vault RNA1-3 is raised and an increased association of vault RNA1-3 with vaults particles has been observed [43, 102]. However, the molecular details behind this observation are still not clear. In general, vault RNAs bound to the vault particles have the capacity to interact with drugs via specific binding sites [103]. For instance, in cancer patients who developed resistance to chemotherapy, the number of vault particles is increased, in agreement with their observed role, in in vitro models [78, 103–105]. Another relevant example is given by mitoxantrone resistance in osteosarcoma, glioblastoma and leukemia where drug failure is based on direct binding of the drug to vtRNA1-1 and vtRNA1-2 [103, 104].

Besides sequestering drugs, vault RNAs are processed into several small RNAs (Fig. 1). Among them small-vault RNAs, account in a second way for multidrug resistance in cancer patients by down-regulating CYP3A4, the key enzyme in drug metabolism [106]. Interestingly, the introduction of 5-methyl-cytosine by the RNA methyltransferase NSUN2-dependent leads to the cleavage of vault RNAs in a Dicer-dependent mechanism; thus, the resulting small-vault RNAs regulate their target genes in a miRNA-like fashion [106–108].

Furthermore, vault RNAs can induce drug resistance in an indirect way by influencing cell proliferation and preventing cell death as described in the following sections.

## Vault RNAs and proliferation

Drug resistance can also arise by the increase in cell proliferation rate [109, 110]. Vault RNAs have been found to influence cell proliferation in different ways and in a cell-type-specific manner without the participation of vault particles (Fig. 1).

In breast cancer, vault RNA1-1 interacts directly with the RNA/DNA-binding protein polypyrimidine tract binding splicing factor (PSF) [105]. PSF is an important regulatory nuclear protein that acts as a component of spliceosomes via the RNA-binding domain and furthermore regulates transcription of genes via the DNA-binding domain; e.g., PSF controls the transcription of P450-linked side-chain cleaving enzyme (CYP11A1) and regulates this by the steroid pathway; in addition PSF inhibits transcription of proto-oncogene G antigen 6 (GAGE6) [111–113]. Following the binding of vault RNA1-1 to PSF RNA-binding domain, the transcriptional repression of GAGE6 via the DNA-binding domain is released and transcription of the proto-oncogene proceeds [114]. Induced expression of GAGE6 results in increased cell proliferation and causes drug resistance [105]. Vault RNA2-1 interacts with and blocks the pro-apoptotic interferon-inducible protein kinase R (PKR). PKR is a central protein for cellular response to different stress signals such as pathogens, starvation, cytokines and irradiation. PKR activates different central pathways like JNK, NF-ϰB, PP2A, p38 and inhibits the eukaryotic translation initiation factor eIF2α by phosphorylation [115]. In normal cells, this inhibits further cellular mRNA translation based on AUG initiation codons and in parallel activates the tumor-suppressor PP2A which blocks cell-cycle, as well as proliferation and leads ultimately to cell death [55]. In different cancer cells, active PKR fails to induce phosphorylation of eIF2α and PP2A, so that apoptosis is not triggered but PKR promotes still the pro-survival NF-ϰB pathway [116–118]. Therefore, the reduced expression levels of vault RNA2-1 found in cancer cell lines and cancer patients specimens result in activation of PKR and subsequent increased cell proliferation as well as drug resistance [119]. Consequently, vault RNA2-1 seems to act as tumor suppressor in contrast to oncogenic effects of vault RNA1-1 [46, 47, 120–123].

## Vault RNAs and apoptosis

Vault RNA1-1 is involved in inhibiting the intrinsic as well as extrinsic apoptosis pathway in several cancer cell lines as demonstrated in in vitro experiments [54, 124]. To address the role of vault RNAs in apoptotic mechanisms, cells have been treated with an autophagy inhibitor and cell death induced by serum starvation. Cells with knock-out vault RNA1-1 gene were more susceptible to programmed cell death; whereas, re-expression of vault RNA1-1 restored apoptosis resistance of the cells. The mechanism underlying the blocking of apoptosis seems to be related to a short stretch within the central domain of vault RNA1-1 and cannot be exerted by other vault RNA members. Furthermore, it was demonstrated that regulation of programmed cell death is independent of vault particles and relay only on vault RNA1-1 [54]. The protective effects of vault RNA1-1 against programmed cell death have been observed after triggering the intrinsic (via staurosporine, etoposide) as well as extrinsic (via Fas ligand) apoptosis pathway [54]. Increased vault RNA1-1 expression activates the pro-survival PI3K-/AKT- and ERK1/2 MAPK-signaling pathways and by this counteract cell death [124]. In Epstein–Barr virus (EBV)-infected B-cells, the latent membrane protein 1 (LMP1) of EBV up-regulates the NF-ϰB pathway that results in increased expression of vault RNA1-1. In this context, vault RNA1-1 inhibits the extrinsic and intrinsic apoptotic pathways and enables cell proliferation by further activation of NF-ϰB pathway and up-regulation of the expression of Bcl-xL [54].

## Vault RNAs and autophagy

Autophagy is besides apoptosis another catabolic pathway essential in homeostasis of cells [125]. Both mechanisms are interconnected by several molecular nodes and a close cross-talk exists [54]. In direct proximity of the VTRNA-1 locus is the proto-cadherin cluster that encodes for the proto-cadherin family, which is involved in autophagy [126]. Therefore, it seems indicative that also vault RNAs might be involved in autophagy [52] (Fig. 1). Autophagic process is necessary for cleaning out unnecessary or dysfunctional components in cells and recycle nutrients and energy. All cargos that cannot be degraded by the ubiquitin–proteasome system are cleaved via autophagy in the lysosomes [127–129]. In contrast to apoptosis that results in cell death [130], autophagy in cancer can facilitate tumor cell survival in stress conditions (e.g., under hypoxia or starving conditions) by providing energy and nutrients [131]. An established marker for the autophagic state of a cell is the intracellular levels of p62 [132]. The selective autophagy receptor p62 [133, 134] is of pivotal importance to the autophagic process by recognizing cargos for the autophagic process, triggering autophagosome formation and exerting a regulatory role in autophagy [127, 135–138]. Vault RNA1-1 binds directly to p62 preventing its oligomerization, a prerequisite for autophagy. This results in inhibition of p62-dependent autophagy and aggregate clearance [52, 139]. Another role of p62 is the cross-talk between autophagy and apoptosis [140, 141] and increased levels of monomeric p62, upon autophagy inhibition via vault RNA1-1, could modulate the balance between the two catabolic pathways. In addition, p62 is involved in the regulation of inflammatory pathways, especially the autophagic defence against invading bacteria and viruses [142]. Most probably, viruses target p62 by up-regulation vault RNAs to decrease the autophagic processes in parallel with inhibition of interferon responses as outlined further below [53].

## Vault RNAs, cellular differentiation and development

It is well established that the levels of non-coding RNAs, including vault RNAs, are highly regulated during development and cellular differentiation since they are essential to these processes [143]. One example is based on the above-mentioned NSUN2-dependent 5-methyl-cytosine modification of vault RNA1.1 and vault RNA1.3 [107, 144] which was recently reported to influence cell differentiation [107, 108]. The serine/arginine-rich splicing factor 2 (SRSF2) binds to the non-methylated form of vault RNA1-1 with higher affinity and counteracts the processing by NSUN2 [108]. Therefore, the expression level of SRSF2 and NSUN2 and their binding to vault RNA1-1 orchestrates the production of small-vault RNAs. The lack of NSUN2-mediated methylation of vault RNA1-1 results in reduced amount of small-vault RNAs and results in changes in epidermal differentiation program of keratinocytes [107, 108]. It is well established also that lack of NSUN2-dependent 5-methyl-cytosine modification in other non-coding RNAs modifies the physiologic situation too; e.g., aberrant 5-methyl-cytosine modification of tRNAs impairs the translation machinery and causes neuro-developmental deficits [145, 146].

The regulated expression of a small-vault RNA derived from vault RNA2-1 (called small-vault RNA2-1a) has been shown to modulate early developmental processes in the central nervous system and has an important role in human brain development as well as aging. The small-vault RNA2-1a has the highest expression level early in post-natal developmental stages and the amount decreases after 1 year with low levels being detected at the oldest ages examined [147].

## Vault RNA-derived small RNAs

Another peculiar characteristic of vault RNAs is that they can be processed into several small RNAs and the cleavage process of vault RNAs is mediated by RNA methyltransferase NSUN2. The introduction of 5-methyl-cytosine by NSUN2 is a prerequisite for DICER-dependent cleavage process of vault RNAs and the resulting small-vault RNAs regulate their target genes in a miRNA-like fashion [106–108] as the aforementioned down-regulation of CYP3A4 by small RNAs resulting in altered dug metabolism [106] as well as the role of small-vault RNAs for epidermal differentiation program of keratinocytes [107, 108]. In both cases, the small-vault RNAs are processed from vault RNA1-1. Another example for the role of small-vault-dependent RNAs was recently reported in prostate cancer. Vault RNA2-1 produces two small RNAs (snc886-3p and snc886-5p) that are found to be reduced in tumor tissues compared to the surrounding normal tissues. Based on PAR-CLIP (photoactivatable ribonucleoside-enhanced crosslinking and immunoprecipitation) and knock-out experiments of microRNA biogenesis enzymes, it was demonstrated that vault RNA2-1 cleavage is based on DICER but independent of DROSHA and the resulting small-vault RNAs are associated with argonaute proteins [148] in a similar process of miRNAs biogenesis [149]. As functional proof of action, over-expression of snc886-3p in relevant in vitro and in vivo systems, resulted in down-regulation of mRNAs containing complementary sequences to the seed sequence of the small-vault RNA in their 3′-UTRs. This led to reduced cell cycle progression, increased apoptosis [148, 150] and this seems in agreement with the view of vault RNA2-1 as tumor suppressor [46, 47, 120–123]. In Parkinson disease, a small-vault RNA derived from vault RNA2-1 is up-regulated in early stages of the disease and this small-vault RNA is most probably involved in the process of brain development as outlined in detail above [151].

Furthermore, small RNAs derived from vault RNAs and associated with the argonaute complex have been identified also in breast, prostate, lung and lymphoid tissue [106, 148]. These findings support the hypothesis of a cleavage of vault RNAs into small RNAs which influence mRNA stability and/or regulate translation like miRNAs. However, the main role of small-vault RNAs need further investigation and it will be of valuable interest if these small RNAs can regulate transcription in a tissue and cell-type-specific fashion as miRNAs [149].

Furthermore, small-vault RNAs are secreted by cells and they are present in high numbers in exosomes (Fig. 1). Therefore, small-vault RNAs are most probably also involved in cell–cell signaling [106, 107, 152].

## Vault RNAs, viral infection and immune system

Viral infections induce vault RNA expression [45, 153] and this was observed in in vitro models for different virus families including γ-herpesviridae (Herpes simplex virus 1), paramyxovirus (Sendai and Epstein–Barr virus), Kaposi's sarcoma-associated herpes and influenza-A virus [44, 45, 53, 54]. Most of these viruses are known to reduce the autophagic capacity of their host cells that is a consequence of high expression levels of vaults RNAs as mentioned above [53, 154]. In addition, transcriptional induction of vault RNAs upon infection, has been associated with expression of latent membrane protein 1 for EBV and non-structural protein NS1 of influenza virus, respectively, with the aim to prevent cells from apoptosis and suppress PKR-mediated innate immunity [53, 54]. Therefore, high expression levels of vault RNAs result in an increased viral load. Viruses are known to hijack cells and their cellular replication machinery to maximize viral replication while inhibiting cellular defence mechanisms [155]. Up-regulation of vault RNA levels seems to be a very efficient way to escape targeted viral degradation via autophagy and subsequent MHC class II antigen presentation [156] and in parallel force the cell to enter a pro-proliferative state that counteracts cellular suicide programs as well as support rapid virus replication [157]. Therefore, it is not surprising that vault RNAs are hijacked and used by viruses. This underlines the important and central role of vault RNAs in regulating cellular processes (Fig. 1).

Another effect of viral infection is the reduction of cellular DUSP11 expression. DUSP11-mediated de-phosphorylation of the 5′-end of vault RNAs initiates the degradation of these RNAs [158, 159]. Therefore, an infection-dependent reduction of DUSP11 levels results in accumulation of vault RNAs that in turn trigger an innate immune response via retinoic acid-inducible gene-1 (RIG-1) receptors [160]. By this, at least one of the anti-viral defence mechanisms against RNA virus is activated [161].

## Vault RNAs as diagnostic and prognostic markers

In Parkinson disease, down-regulation of miR-7, miR-34b/c and miR-133b [162–164] as well as up-regulation of a small-vault RNA derived from vault RNA2-1 is common in brain areas that are affected by this disease [147]. Increased expression of vault RNA2-1 occurs early in the course of disease and could perhaps be used as a diagnostic marker.

Hyper-methylation of vault RNA2-1 gene is correlated with poor prognosis and overall survival in several cancers; e.g., gastric, oesophageal, lung, prostate, acute myeloid leukemia and myelodysplastic syndrome. Therefore, vault RNA2-1 could act as tumor suppressor [46, 47, 120–123, 150] and the expression level of vault RNA2-1 could be used as a prognostic marker.

In addition, the expression level of the major vault protein has been correlated with therapy resistance, prognosis and overall survival in several blood cancers (acute myeloid leukemia, acute lymphoblastic leukemia, adult T-cellss leukemia and multiple myeloma) [165–176]. In solid tumors, expression level of the major vault protein is a good prediction factor for response to chemotherapy in bladder cancer [177], melanoma [178] and for determining the aggressive phenotype of testicular germ-cell tumors [179] and glioblastoma [180].

## Conclusion and perspectives

The old simplistic view that non-coding RNAs only play functional roles in protein synthesis as integral components (rRNA) or reaction substrates (tRNA) of the ribosome has dramatically evolved during the last 2 decades with emerging concepts linking different classes of non-coding RNAs to physiology and disease. The non-coding RNA group of vault RNAs, which is composed of only four members in human, exert an important role within the cell. Although until recently not all functions and processes have been unveiled in detail, it is already clear that vault RNAs add another level of regulation to the network of non-coding and coding RNAs. As outlined in this review, vault RNAs are involved in transferring extracellular stimuli into signals inside the cell; they regulate central signaling pathways and cell–cell communication. Furthermore, vault RNAs play a substantial role in immunity response, influencing proliferation, apoptosis and autophagy as well as being involved in drug resistance mechanisms (Fig. 1). All these functions are under vault RNAs regulation either via direct interaction with proteins or via post-transcriptional regulation of mRNAs. In particular, in the context of cancer, vault RNAs appear to have a critical role and a better understanding of their biology in this disease could offer a new prospect for cancer treatment and prevention of drug resistance.
